# Endocannabinoids Released in the Ventral Tegmental Area During Copulation to Satiety Modulate Changes in Glutamate Receptors Associated With Synaptic Plasticity Processes

**DOI:** 10.3389/fnsyn.2021.701290

**Published:** 2021-08-18

**Authors:** Gabriela Rodríguez-Manzo, Estefanía González-Morales, René Garduño-Gutiérrez

**Affiliations:** Departamento de Farmacobiología, Centro de Investigación y de Estudios Avanzados (Cinvestav-Sede Sur), Ciudad de México, Mexico

**Keywords:** endocannabinoids, CB1 receptors, AMPA and NMDA glutamate receptors, mesolimbic circuit, ventral tegmental area, synaptic plasticity, sexual satiety, natural reward

## Abstract

Endocannabinoids modulate mesolimbic (MSL) dopamine (DA) neurons firing at the ventral tegmental area (VTA). These neurons are activated by copulation, increasing DA release in nucleus accumbens (NAcc). Copulation to satiety in male rats implies repeated ejaculation within a short period (around 2.5 h), during which NAcc dopamine concentrations remain elevated, suggesting continuous neuronal activation. During the 72 h that follow copulation to satiety, males exhibit long-lasting changes suggestive of brain plasticity processes. Enhanced DA neuron activity triggers the synthesis and release of endocannabinoids (eCBs) in the VTA, which participate in several long-term synaptic plasticity processes. Blockade of cannabinoid type 1 receptors (CB1Rs) during copulation to satiety interferes with the appearance of the plastic changes. Glutamatergic inputs to the VTA express CB1Rs and contribute to DA neuron burst firing and synaptic plasticity. We hypothesized that eCBs, released during copulation to satiety, would activate VTA CB1Rs and modulate synaptic plasticity processes involving glutamatergic transmission. To test this hypothesis, we determined changes in VTA CB1R density, phosphorylation, and internalization in rats that copulated to satiety 24 h earlier as compared both to animals that ejaculated only once and to sexually experienced unmated males. Changes in glutamate AMPAR and NMDAR densities and subunit composition and in ERK1/2 activation were determined in the VTA of males that copulated to satiety in the presence or absence of AM251, a CB1R antagonist. The CB1R density decreased and the proportion of phosphorylated CB1Rs increased in the animals that copulated compared to control rats. The CB1R internalization was detected only in sexually satiated males. A decrease in α-amino-3-hydroxy-5-methylisoxazole-4-propionate receptor (AMPAR) density, blocked by AM251 pretreatment, and an increase in the proportion of GluA2-AMPARs occurred in sexually satiated rats. GluN2A- N-methyl-D-aspartate receptor (NMDAR) expression decreased, and GluN2B-NMDARs increased in these animals, both of which were prevented by AM251 pre-treatment. An increase in phosphorylated ERK1/2 emerged in males copulating to satiety in the presence of AM251. Results demonstrate that during copulation to satiety, eCBs activate CB1Rs in the VTA, producing changes in glutamate receptors compatible with a reduced neuronal activation. These changes could play a role in the induction of the long-lasting physiological changes that characterize sexually satiated rats.

## Introduction

The mesolimbic (MSL) dopaminergic system is constituted by the dopaminergic neurons of the ventral tegmental area (VTA) that mainly project to the nucleus accumbens (NAcc) (Ikemoto, 2007). This circuit is activated by natural rewarding behaviors, such as sexual activity (Kelley and Berridge, 2002), increasing dopamine (DA) levels in the NAcc. Such DA increases occur after the exposure of male rats to estrous odors, to an inaccessible sexually receptive female, and in response to mating itself (Pfaus et al., 1990; Damsma et al., 1992; Mitchell and Gratton, 1994; Robinson et al., 2001), and are the result of the activation of VTA neurons. This has been demonstrated in studies showing that sex-related cues and copulation increase the expression of c-Fos, a marker of neuronal activity, in the DA neurons of the VTA (Balfour et al., 2006) and that the sexual behavior performance increases VTA neuronal firing (Hernandez-Gonzalez et al., 1997) and activates NAcc medium spiny neurons (MSNs) (Matsumoto et al., 2012). Conversely, the MSL system has been shown to participate in the regulation of sexual motivation and the reinforcing properties of copulatory behavior (Everitt, 1990; Melis and Argiolas, 1995; Pfaus, 2009).

Sexually experienced male rats that are allowed to copulate without restriction with a sexually receptive female will repeatedly ejaculate until becoming sexually satiated. Intense copulation is displayed along 2.5 h, time during which male rats are capable of executing 7 successive ejaculations, on an average, before becoming sexually inhibited. Interestingly, this sexual inhibition lasts 72 h, but the sexually satiated males require 15 days of sexual inactivity to fully recover their initial ejaculatory capacity (Rodríguez-Manzo and Fernández-Guasti, 1994; Rodríguez-Manzo et al., 2011). The duration of this sexual inhibitory state suggests the occurrence of neuroadaptations resulting from intense copulation within a short period. In support of this idea, the sexually satiated rats exhibit other physiological changes in addition to the sexual behavior inhibition, the most conspicuous of which is a generalized sensitization to drug actions, a phenomenon which also lasts 72 h (Rodríguez-Manzo et al., 2011).

It has been demonstrated that during the copulation to exhaustion process, the NAcc DA levels are continuously elevated (Fiorino et al., 1997), indicating the constant activation of MSL DA neurons. Enhanced midbrain DA neuron activity triggers the synthesis and release of endocannabinoids (eCBs) from their cell bodies in the VTA (Lupica and Riegel, 2005). These eCBs act retrogradely, activating presynaptic cannabinoid type 1 receptors (CB1Rs) located on GABAergic (Szabo et al., 2002) and glutamatergic axon terminals (Melis, 2004), where they regulate neurotransmitter release, thereby modulating the firing activity of midbrain DA neurons (Riegel and Lupica, 2004). This eCB-mediated regulation is exerted through the transient inhibition of neurotransmitter release by the following two short-term plasticity phenomena: depolarization-induced suppression of inhibition (DSI), when inhibiting GABA release (Ohno-Shosaku et al., 2001; Wilson and Nicoll, 2001), and depolarization-induced suppression of excitation (DSE), when inhibiting glutamate release (Kreitzer and Regehr, 2001; Melis, 2004). The eCBs are also involved in the induction of long-term forms of synaptic plasticity, namely long-term depression (eCB-LTD) at both, glutamatergic (Gerdeman et al., 2002) and GABAergic synapses (Chevaleyre and Castillo, 2003). Besides, glutamatergic transmission at the VTA has been implicated in the induction of DA neuron burst firing, responsible for massive synaptic DA release in the NAcc (Floresco et al., 2003), as well as in long-term synaptic plasticity phenomena at the VTA (Xin et al., 2016). VTA neurons that are activated by copulation receive glutamatergic inputs (Balfour et al., 2006). Plasticity phenomena of excitatory synaptic transmission involve changes in α-amino-3-hydroxy-5-methylisoxazole-4-propionate (AMPA) and N-methyl-D-aspartate (NMDA) receptor densities and subunit composition (Song and Huganir, 2002; Paoletti et al., 2013).

Previous work from our laboratory showed that the blockade of CB1Rs during copulation to satiety, interferes with the appearance of the long-lasting sexual behavior inhibition and drug hypersensitivity in the sexually satiated rats (Canseco-Alba and Rodríguez-Manzo, 2014; González-Morales and Rodríguez-Manzo, 2020), evidencing the involvement of eCBs in the induction of these phenomena. These effects were replicated by the specific blockade of CB1Rs at the VTA (Canseco-Alba and Rodríguez-Manzo, 2016). On these bases, we hypothesized that eCBs released at the VTA during copulation to satiety could induce synaptic plasticity processes through the activation of CB1Rs that might involve glutamatergic transmission. To test this hypothesis, in the present work, we first determined if there were changes in VTA CB1R density, phosphorylation, and internalization—all events indicative of CB1R activation—in sexually experienced unmated control males and in rats that ejaculated only once or copulated to satiety 24 h earlier. In addition, we established the occurrence of changes in glutamate AMPA receptor (AMPAR) and NMDA receptor (NMDAR) densities and subunit composition in the VTA of male rats that copulated to satiety in the absence or presence of the CB1R antagonist, AM251. Finally, we explored if there were changes in the phosphorylation of the extracellular signal-regulated kinase (pERK 1/2), which has been associated with CB1R activation and to long-term synaptic plasticity processes.

## Materials and Methods

### Animals

Sexually experienced, adult male (250–300 g b. wt.) and sexually receptive female (200 g b.wt.) Wistar rats from our own vivarium were used in this study. Animals were housed, eight per cage, under inverted light/dark cycle conditions (12 h light: 12 h dark, lights on at 22:00 h), at 22°C, and with free access to food and water. Males were rendered sexually experienced by subjecting them to five independent sexual behavior tests and those animals achieving ejaculation in <15 min, in at least three of these tests, were classified as sexually experienced and selected for the study. Female rats served as sexual stimuli; sexual receptivity was induced by the sequential s.c. injection of estradiol benzoate (13 μg/rat) followed 24 h later by progesterone (7 mg/rat). All experimental procedures (Protocol 0230-16) were approved by our Institutional Internal Committee for the Care and Use of Laboratory Animals (Comité Institucional para el Cuidado y Uso de Animales de Laboratorio, CICUAL), which follows the regulations established in the Mexican Official Norm for the use and care of laboratory animals NOM-062-ZOO-1999.

### Sexual Behavior and Sexual Satiety Tests

Sexual behavior tests were conducted 2 h after the beginning of the dark phase of the cycle, in a room under dim red light. Males were individually introduced into polycarbonate cylindrical arenas covered with fine sawdust. A 5-min adaptation period was permitted to the males before introducing a sexually receptive female. Sexual activity was allowed until the achievement of one ejaculation or the attainment of sexual satiety. In this last case, males had *ad libitum* copulation with a single sexually receptive female until a 90-min period elapsed since the last ejaculation, without accomplishment of another ejaculation, which is the criterion used to consider a male sexually satiated (Rodríguez-Manzo et al., 2011). After reaching the corresponding criteria, animals were returned to their home cages.

### Drugs

All drugs were purchased from Sigma-Aldrich (St. Louis, MO, USA). AM251 was dissolved in a mixture of saline solution (98%), Tween80 (1%), and dimethyl sulfoxide (DMSO, 1%) and was i.p. injected. Estradiol benzoate and progesterone were dissolved in sesame oil and s.c. injected.

### Antibodies

The following primary antibodies were used: rabbit anti-CB1R, mouse anti-β-actin, goat anti-β-Arrestin2 (β-A2), mouse anti-GluN2B and mouse anti-GLUA2 (Santa Cruz Biotechnology, Dallas, TX, USA); rabbit anti-GluN2A, rabbit anti-GluN1and rabbit anti-GluA1 (Alomone Labs, Jerusalem, Israel); rabbit anti-CB1R phospho S316 (pCB1R) (Abcam, Cambridge, UK), rabbit anti-ERK 1/2 phospho Thr202/Tyr204 (pERK 1/2) (Cell Signaling Technology, Danvers, MA, USA) and mouse anti-VGlut2 (Merck-Millipore, Burlington, MA, USA). Secondary Abs for immunoblotting include HRP-donkey anti-rabbit and HRP-goat anti-mouse (Jackson ImmunoResearch, West Grove, PA, USA). For immunofluorescence, the secondary Abs used were donkey anti-goat Alexa Fluor 488, donkey anti-mouse Alexa Fluor 555, and donkey anti-rabbit Alexa Fluor 647, all from Invitrogen (Thermo Fisher Scientific, San Diego, CA, USA).

### Western Blot

Twenty-four hours after the sexual behavior tests, the animals were anesthetized with sodium pentobarbital and thereafter sacrificed by decapitation, their brains extracted in <5 min and cooled in dry ice for 15 s. Using stereotaxic coordinates from a rat brain atlas (Paxinos and Watson, 2009) as a reference, 2 mm thick coronal slices from the region containing the VTA were obtained. Bilateral punches of the VTA (1 mm diameter) from each rat were obtained with the aid of sample punches (Fine Science Tools Inc., CA, USA). Samples of both hemispheres of each rat were pooled, put in a lysis buffer [Tris HCL (50 mM), NaCl (150 mM), Igepal (1%), Triton X-100 (1%)] containing protein phosphatase (Thermo Fisher, catalog # 78428, 100X) and protease (Thermo Fisher, catalog # 78430,100X) inhibitor cocktails, pH = 7.4 and stored at −80°C until further processing. Tissue samples were thawed, homogenized by sonication, and centrifuged at 14,000 rpm, for 30 min, at 4°C; the resulting pellet was removed and protein concentrations were quantified in the supernatant using the modified Lowry method (DC Protein assay instruction manual, BIORAD (Lowry et al., 1951; Peterson, 1979). Bovine serum albumin (BSA, Sigma catalog # 05470) was used to build protein reference standard curves.

From each tissue sample, 25 μg protein, diluted 1:1 in 2X loading buffer (Sigma, catalog # S3401) were boiled to 95°C for 5 min, loaded onto 8% SDS-polyacrylamide gels and separated by electrophoresis for 2 h, at 100 V. A protein molecular weight (MW) standard (Page ruler plus pre-stained protein ladder, Thermo Fisher, catalog # 26620) was added to each gel. Proteins were electrophoretically transferred onto a polyvinylidene fluoride (PVDF) membrane for 1 h, at 100 V, 350 mA. PVDF membranes were blocked for 2.5 h, at room temperature, with 4% milk (Biorad, catalog # 1706404) in Tris-Buffered Saline (TBS) with 1% Tween20 (Sigma, catalog # P9416) (TBS-T) for CB1R and pERK 1/2 Abs, and with 2% milk for the rest of Abs used (GluN1, GluN2A, GluN2B, GluA1, GluA2, and pCB1R). Thereafter, the membranes were incubated with the primary antibody overnight, at 4°C. Membranes were then washed three times with TBS-T for 5–10 min, under agitation, and were incubated with the corresponding secondary antibody for 1.5 h at room temperature. After this, they were washed three times with TBS-T and once with TBS. A chemiluminescence kit (Millipore, catalog #WBKLS0500) was used to reveal the membranes using the BioradChemidoc MP Imaging system (Hercules, CA, USA). Optical density (OD) of the resulting bands was determined with the aid of the ImageJ program (NIH) and the values were normalized with those of β-actin, which served as a charge control. Details on the Abs used for Western blotting are summarized in Table 1.

**Table 1:** Summary of antibodies used for the Western blotting.

	**Primary antibodies**				**Secondary antibodies**		
**Protein**	**Source**	**Dilution**	**Catalog #**	**References**	**Source**	**Dilution**	**Catalog #**
**Western blot**							
CB1	Rabbit polyclonal	1:1200	sc-20754	Almada et al., 2016	Donkey anti-rabbit	1:10000	711-035-152
pCB1	Rabbit monoclonal	1:1000	ab186428				
GLUN1 (NMDA)	Rabbit polyclonal	1:700	AGC-001	Atkin et al., 2015			
GLUN2A (NMDA)	Rabbit polyclonal	1:700	AGC-002	Atkin et al., 2015			
GLUN2B (NMDA)	Mouse monoclonal	1:1000	sc-365597	Lai et al., 2019	Goat anti-mouse	1:10000	115-035-003
GluA1 (AMPA)	Rabbit polyclonal	1:600	AGC-004	Lin et al., 2011	Donkey anti-rabbit	1:10000	711-035-152
GluA2 (AMPA)	Mouse monoclonal	1:500	sc-517265	Scherma et al., 2020	Goat anti-mouse	1:10000	115-035-003
pERK1/2(Thr202/Tyr204)	Rabbit polyclonal	1:10000	9101	Travaglia et al., 2016	Donkey anti-rabbit	1:10000	711-035-152
β-Actin	Mouse monoclonal	1:10000	sc-47778	Xu et al., 2020	Goat anti-mouse	1:10000	115.035-003

### Immunofluorescence for CB1R and β-A2

Double immunofluorescence and confocal microscopy were used to analyze CB1R co-localization with β-arrestin2 (βA2), as a tool to determine CB1 cannabinoid receptor internalization in the VTA of male rats that ejaculated once or copulated to satiety 24 h earlier.

#### Tissue Preparation

Twenty-four hours after the sexual behavior tests, animals were deeply anesthetized with sodium pentobarbital (200 mg/kg, i.p.) and intracardially perfused with 250 ml 0.9% sodium chloride, followed by 400 ml paraformaldehyde (4%) in sodium phosphate solution (PBS) buffer 0.1 M supplemented with 0.9% sodium chloride, pH 7.3. At the end of this procedure, brains were extracted from the skull and immediately placed in a cryoprotectant solution (sucrose at 30% in PBS) and stored at 4°C until further use.

Sixty coronal sections (40 μm) of the brain region containing the VTA [coordinates from −5.2 to −6.3 mm anterior to bregma (Paxinos and Watson, 2009)] were obtained using a cryostat (Leica CM1100, Nussloch, Germany); 10 brain sections from each animal, representative of the whole VTA, were used for the immunofluorescence experiments.

#### Triple Immunofluorescence for CB1R, β-A2, and VGlut2

Tissue sections were washed with PBS, four times, for 10 min and incubated during 2.5 h with a blocking solution [2.0% BSA, (Sigma, catalog # 05470), 6% donkey serum (Sigma, catalog # D9663), 2% gelatin (Sigma, catalog # G7765), and 0.3% Triton X-100 (Sigma, catalog # T9284), all contained in PBS]. All incubations and washes were made under constant stirring. Thereafter, sections were incubated with the CB1R, β-A2, and VGlut2 primary antibodies, diluted in the blocking solution, first for 2 h at room temperature, then for 19 h at 4°C and finally, 2 h at room temperature. After incubations with the primary antibodies, the sections were washed four times, for 10 min each, with a solution containing 0.3% Triton X-100 and 2.0% gelatin mixed in PBS. Subsequently, they were incubated with the secondary antibodies and 1 μl DAPI (Sigma catalog # D8417), diluted in the blocking solution, for 2 h at room temperature. For immunohistochemical controls, the first antibodies were omitted, which resulted in the absence of immunoreactivity. After incubation, the slices were washed four times with 0.1% Triton X-100 in PBS, for 10 min each. Thereafter, the sections were mounted on gelatinized slides using antifade mounting medium (Prolong Diamond antifade kit, Molecular Probes catalog # P36962), and analyzed by confocal microscopy. Details on the Abs used for immunohistochemistry procedures are summarized in Table 2.

**Table 2:** Summary of antibodies used for immunohistochemical procedures.

	**Primary antibodies**				**Secondary antibodies**		
**Protein**	**Source**	**Dilution**	**Catalog #**	**References**	**Source**	**Dilution**	**Catalog #**
**Immunohistochemistry**							
CB1	Rabbit polyclonal	1:75	sc-20754	Hayn et al., 2008	Donkey anti-rabbit, Alexa 647	1:175	A31573
β-Arrestin2	Goat polyclonal	1:75	sc-6387	Garduño-Gutiérrez et al., 2013	Donkey anti-goat, Alexa 488	1:250	A11055
VGlut2	Mouse monoclonal	1:330	MAB5504	Zhang et al., 2015	Donkey anti-mouse, Alexa 555	1:300	A31750

#### Confocal Analysis

Ten coronal brain sections, representative of the whole VTA, of three different rats from each group were used for CB1R and β-A2 immunodetection. The VTA region in these sections was localized with the 10X objective of a confocal microscope (Carl Zeiss, LSM 800 Airyscan). Once identified, the objective was changed to a 40x oil objective for image acquisition. The ZEN v10.0 black software was used for image acquisition and the ZEN 2.3 blue edition software for image processing (Carl Zeiss, Oberkochen, Germany). Images were acquired in the best signal mode with a 1 airy unit (AU) pinhole and the total optical area for the 40x objective was 24,336 μm^2^. The maximum emission spectrum of each fluorophore was delimited with barrier filters to avoid crosstalk between spectral emission curves. The 450 nm laser was used to detect DAPI, the 488 nm laser to detect Alexa 488, the 555 nm laser to detect Alexa 555, and the 647 nm laser to detect Alexa 647. The final color of each fluorophore (secondary antibodies) in the images was selected from the image software: magenta for Alexa 647 (CB1R), red for Alexa 555 (VGlut2), and green for Alexa 488 (β-A2). The immunoreactivities (IRs) for CB1R and β-A2 and their co-localization were obtained from three different channels (channel 1: Alexa 488 for β-A2, channel 2: Alexa 647 for CB1R, and channel 3: CB1R-IR/β-A2-IR co-localization). The IR values were obtained in μm^2^, which were provided by the ZEN blue software.

#### Quantitative Analysis

For the quantification of the CB1R and β-A2 densities, data were expressed as the ratio of each IR protein area/total analyzed area (24,336 μm^2^), using the following formulae: CB1R-IR = [channel 1 + channel 3 (μm^2^)]/24,336 μm^2^; β-A2-IR = [channel 2 + channel 3 (μm^2^)]/24,336 μm^2^. CB1R-IR/β-A2-IR co-localization was considered indicative of receptor internalization (Garduño-Gutiérrez et al., 2013). The degree of CB1R internalization was determined dividing IR co-localization values by the total CB1R-IR using the following formula: [channel 3 (co-localization of CB1R-IR and β-A2-IR) (μm^2^)]/[channel 1 (CB1R-IR area) + channel 3 (co-localization of CB1R-IR and β-A2-IR) (μm^2^)]. Data of a total of 30 brain sections per group were used for statistical analysis.

### Statistical Analyses

Data are expressed as mean ± standard error of the mean (SEM). Changes in protein expression (CB1R, pCB1R, GluA1, GluA2, GluN1, GluN2A, GluN2B, and pERK1/2) were conducted by means of a one-way ANOVA followed by Tukey's test. Paired comparisons were conducted with the unpaired *t*-test; when normality was not met, the Mann–Whitney U test was used. Immunohistochemistry data were not normally distributed and were therefore analyzed by means of the Kruskal–Wallis ANOVA followed by Dunn's test. The Sigma Plot program, version 12.0 was used for all statistical analyses. In all cases, differences with a *P* ≤ 0.05 were considered significant.

### Experimental Design

Three different groups of males were used to establish possible changes in CB1R and pCB1R densities (*n* = 5 each) as well as in CB1R internalization (*n* = 3): (1) sexually experienced males that had no sexual activity 1 week prior to sacrifice, which served as a control group; (2) sexually experienced males that ejaculated once and were sacrificed 24 h later, which were used as a reference for the effects of recent sexual activity; and (3) sexually experienced males that copulated to satiety and were sacrificed 24 h later, which constituted the group of interest.

For the determination of changes in the densities of AMPARs (GluA1 and GLUA2 subunits), NMDARs (GluN1, GluN2A, and GluN2B subunits) and the phosphorylated MAPK ERK1/2 (pERK1/2), a fourth group was included (*n* = 5). This group consisted of sexually experienced males that copulated to satiety in the presence of the CB1R antagonist, AM251 (0.3 mg/kg, i.p.), administered immediately before the copulation to satiety session, at a dose previously found to interfere with the appearance, 24 h later, of the long-lasting sexual behavior inhibition and drug hypersensitivity that characterize sexually satiated male rats (González-Morales and Rodríguez-Manzo, 2020). These animals were sacrificed 24 h later to find out if eCBs played a role in the putative changes in these proteins in the sexually satiated males. For the WB experiments, animals were anesthetized with sodium pentobarbital and sacrificed by decapitation to obtain the brain samples. For the immunohistochemical experiments, the animals were deeply anesthetized with sodium pentobarbital (200 mg/kg, i.p.), and intracardially perfused.

### Experiment 1. Evidence for CB1R Activation in the VTA by Copulation to Satiety

In each of the three groups of males, the density of CB1Rs, as well as changes in the density of phosphorylated CB1Rs, were determined by Western blot. In addition, immunohistochemistry and confocal microscopy were used to establish CB1R co-localization with β-A2, as an indicator of CB1R internalization (Garduño-Gutiérrez et al., 2013).

### Experiment 2. Determination of Changes in AMPARs of the VTA Induced by Copulation to Satiety and the Possible Involvement of eCBs in Their Induction

The density of VTA AMPARs was established in each of the four groups of males, by determining the expression of the constitutive GluA1 subunit of these receptors.

The expression of AMPARs containing the GluA2 subunit was also established in each of these groups, establishing the density of the GluA2 subunit. A Western blot procedure was used for all determinations.

### Experiment 3. Establishment of Changes in NMDARs of the VTA Induced by Copulation to Satiety and the Possible Involvement of eCBs in Their Induction

The density of VTA NMDARs was established in the same four groups of animals, by determining the expression of the obligatory, channel-forming GluN1 subunit of these receptors. In addition, the densities of NMDARs containing the GluN2A subunit and of those containing the GluN2B subunit in all the four groups were established by determining the expression of the GluN2A or the GluN2B subunit. All determinations were carried on by Western blot.

### Experiment 4. Establishment of Changes in VTA pERK1/2 Densities Induced by Copulation to Satiety and the Possible Involvement of eCBs in Their Induction

The expression of the phosphorylated form of the MAPK ERK1/2 (pERK1/2) was determined by Western blot in each of the four different groups of animals.

## Results

### Evidence for CB1R Activation in the VTA by Copulation to Satiety

**Figure 1:**
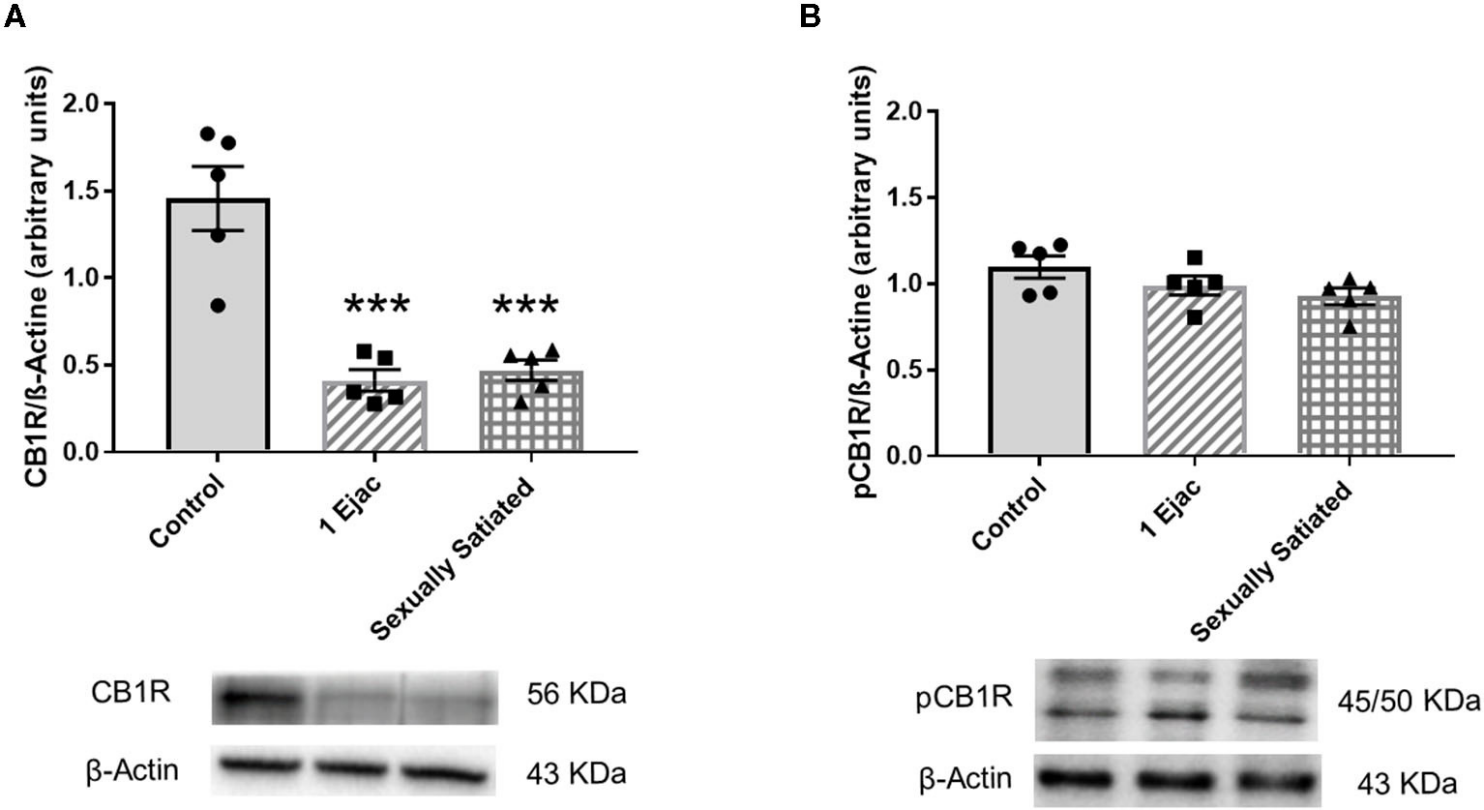
Changes in the densities of CB1R and pCB1R in the VTA of male rats in response to copulation. Western blot analysis showing changes in CB1R **(A)** and phosphorylated CB1R (pCB1R) **(B)** densities in the VTA of males that ejaculated once (1 Ejac) and males that copulated to satiety (Sexually satiated) 24 h earlier when compared to control sexually experienced unmated rats (Control). Values are mean ± S.E.M of the protein/β-Actin optical density (O.D.) ratios of 5 rats per group. One-way ANOVA followed by Tukey test, ^***^P < 0.001 vs. control.

Changes in VTA CB1R density produced by sexual activity are shown in Figure 1. There was a significant effect of mating on CB1R expression [one-way ANOVA, *F*_(2, 12)_ = 25.20, *P* < 0.001]. *Post-hoc* analysis showed a significant decrease in the density of CB1Rs (Figure 1A) in both, the animals that executed one ejaculatory series (1 Ejac) and those that copulated to satiety (sexually satiated) the day before sacrifice, as compared to unmated sexually experienced control males (Tukey's test, *P* < 0.001 each). By contrast, as shown in Figure 1B, no significant differences in the expression of phosphorylated CB1Rs (pCB1R) were found among groups [one-way ANOVA, *F*_(2, 12)_ = 2.33, *P* = 0.14].

**Figure 2:**
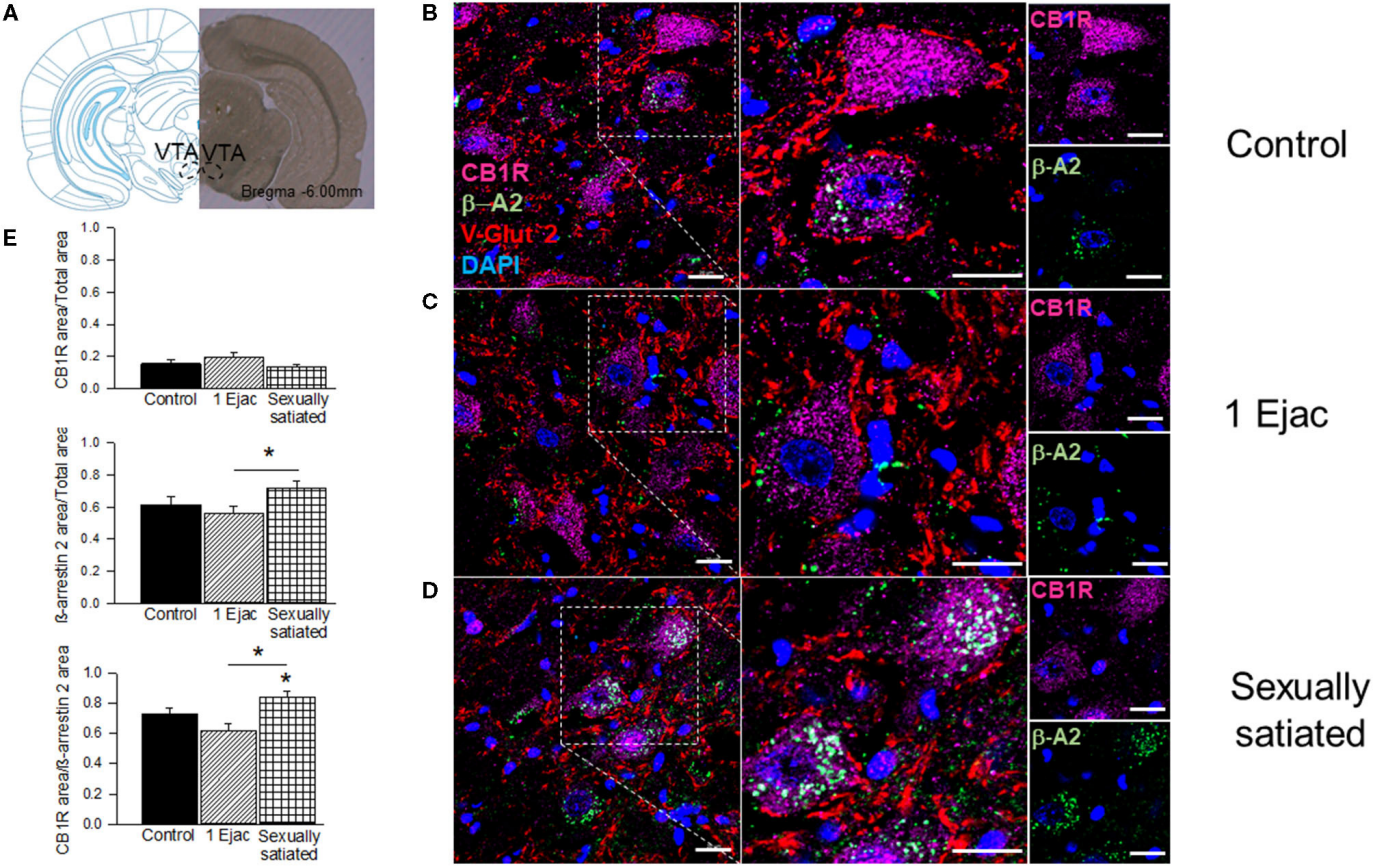
CB1R internalization in the VTA of male rats that copulated to satiety. **(A)** Shows a coronal brain section, taken at −6.00 mm anterior to bregma, showing the VTA region from where the sample photomicrographs were obtained. **(B–D)** Show representative confocal photomicrographs from the VTA of unmated sexually experienced male rats (Control), males that ejaculated once (1 Ejac) and males that copulated to satiety (Sexually satiated) 24 h earlier, respectively. The first column shows 40X magnification images of CB1R (Alexa 647, magenta), β-A2 (Alexa 488, green) and VGlut2 (Alexa 555, red) immunoreactivity (IR); cell nuclei appear in blue (DAPI). The second column depicts a 2x enlargement of the selected region with the same IR marks. The third column shows individual CB1R and β-A2 IR-marks in the selected region. Co-localization of CB1R with β-A2- appears in white color. Scale bar 20 μm. **(E)** Shows the quantification of CB1R-IR, β-A2-IR as well as that of their co-localization. Kruskal-Wallis followed by Dunn's test, ^*^P < 0.05.

Immunohistochemical and confocal microscopy analyses of mating-induced CB1R internalization is shown in Figure 2. Sample photomicrographs of the VTA, taken at the level of −6.0 mm from bregma (Figure 2A), were included in Figure 2. The upper row of photomicrographs corresponds to control, sexually experienced unmated rats (Figure 2B), the middle row refers to males that ejaculated once (Figure 2C), and the lower row to sexually satiated animals (Figure 2D). Similar patterns of CB1R immunoreactivity (CB1R-IR, magenta) distribution can be observed in all groups, i.e., a substantial amount of CB1R-IR could be detected, which was mainly located intracellularly, uniformly distributed in the cytoplasm of cell bodies with a defined nucleus (DAPI, blue). In addition, CB1R-IR appeared as punctuate marks, outside these rounded structures, that could indicate their presence in fibers. Quantification of CB1R-IR (Figure 2E) did not show statistically significant differences among groups [Kruskal–Wallis ANOVA, *H*_(2, 30)_ = 3.76, *P* = 0.15].

Ventral tegmental area β-A2 immunofluorescence (β-A2-IR, green) can be detected in all groups. In the control group (Figure 2B), β-A2-IR appears mainly as punctuating marks, sometimes forming empty circles, and occasionally distributed in the cytoplasm of cell bodies with a defined nucleus (DAPI, blue color). In the samples of males that ejaculated once (Figure 2C), β-A2-IR appears sometimes forming empty circles, which might correspond to cell body membranes. Finally, in the sexually satiated males (Figure 2D), an increase in β-A2-IR, together with a clear redistribution of the immunoreactive mark can be appreciated. In this case, clusters of β-A2-IR can be observed predominantly in the cytoplasm of cell bodies with a defined nucleus. However, punctuate marks can also be seen independently of these cytoplasmic clusters. Quantification of β-A2-IR evidenced statistically significant differences in its expression among groups [Kruskal–Wallis ANOVA, *H*_(2, 30)_ = 6.89, *P* = 0.03]. *Post-hoc* analysis indicated a statistically significant increase in β-A2-IR in the VTA of the sexually satiated males when compared to animals that ejaculated only once (Dunn's test *P* < 0.05). The first two columns of the sample photomicrographs show the co-localization of CB1Rs with the β-A2 protein, which combined immunofluorescences produced a white color. Higher magnification images (2x) are included in the second column. Colocalization sample photomicrographs contain an additional immunoreactive mark for the glutamate vesicular transporter 2 (VGlut2-IR, red color), which presumably labels glutamatergic inputs to the VTA of subcortical origin, which are the most abundant (Omelchenko and Sesack, 2007). In the photomicrographs of control males, CB1R-IR/β-A2-IR colocalization is almost absent. In this sample image, the coincidence of both immunoreactive marks is only observed in the cytoplasm of one cell. VGlut2-IR is abundant and located mainly outside cell bodies; sometimes this mark coincides with the cell membrane, but the majority of VGlut2-IR can be seen in what appears to represent fibers. The sample photomicrograph of males that ejaculated once lacks CB1R-IR/β-A2-IR colocalization and the majority of VGlut2-IR is observed again in fibers. Finally, in the sample of the sexually satiated males CB1R-IR/β-A2-IR co-localization is evident in the cytoplasm of cell bodies with a defined nucleus, suggesting CB1R internalization. VGlut2-IR appears often in the vicinity of the cells showing CB1R-IR/β-A2-IR colocalization. Quantification of CB1R/β-A2 colocalization showed differences among groups [Kruskal–Wallis ANOVA, *H*_(2, 30)_ = 15.10, *P* < 0.001]. A statistically significant increase in the colocalization of both marks was obtained in the sexually satiated males as compared to control unmated males and to animals ejaculating once (Dunn's test, *P* < 0.05 each).

### Changes in AMPARs of the VTA Induced by Copulation to Satiety and Involvement of eCBs in Their Induction

**Figure 3:**
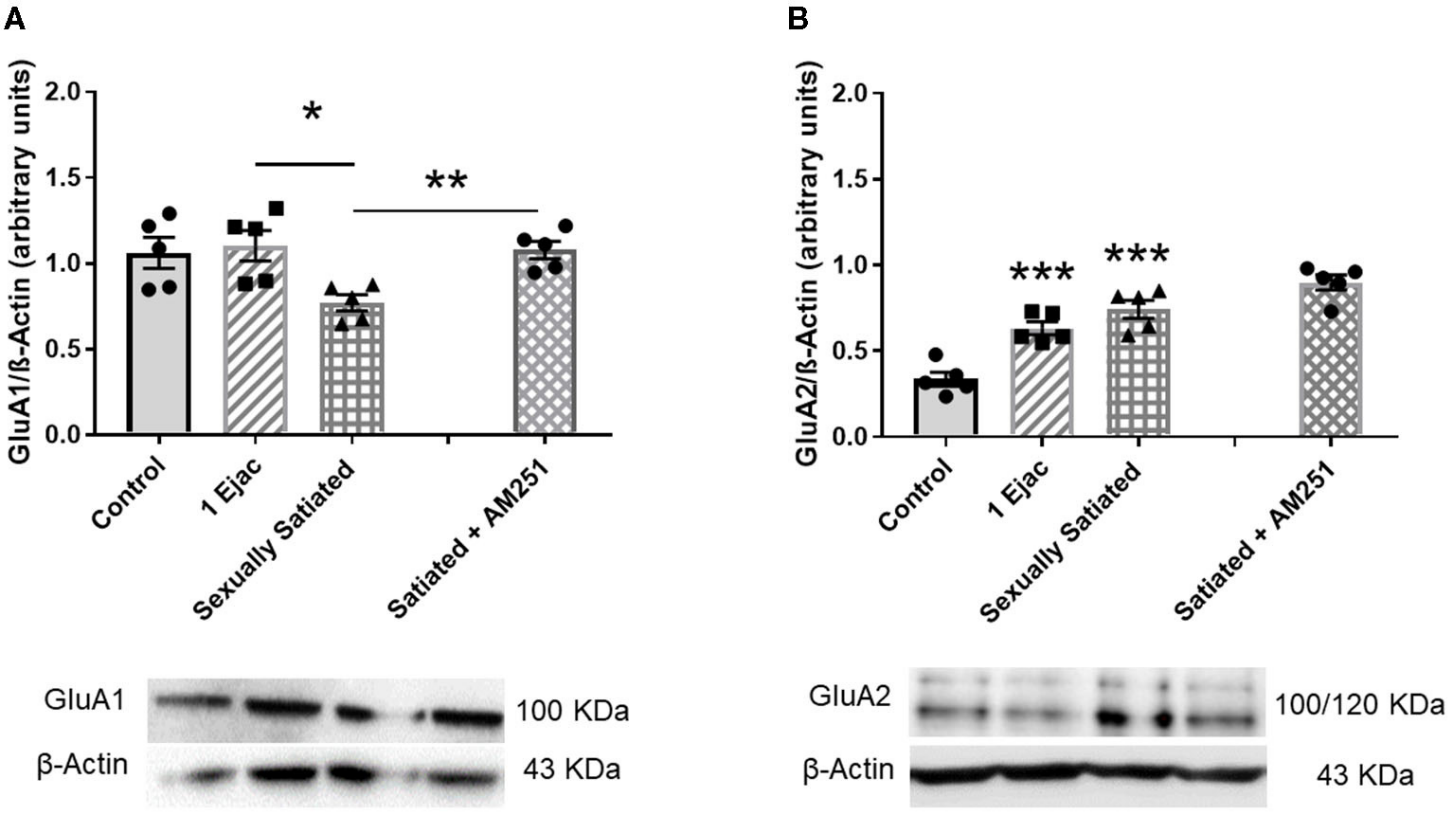
Changes in AMPAR density and subunit composition in the VTA of sexually satiated rats and involvement of eCBs in their induction. Western blot analysis showing changes in GluA1 **(A)** and GluA2 AMPAR subunit **(B)** densities in the VTA of control sexually experienced unmated rats (Control), males that ejaculated once (1 Ejac) or copulated to satiety (Sexually satiated) 24 h earlier, and of males that copulated to satiety in the presence of the CB1R antagonist AM251 (Satiated + AM251). Differences among untreated groups with different sexual conditions were determined by means of a one-way ANOVA followed by Tukey test, ^***^P < 0.001; ^*^P < 0.05. A comparison between the rats that copulated to satiety in the absence or presence of AM251 was conducted with the unpaired *t*-test ^**^P < 0.01. Values are mean ± S.E.M of the protein/β-Actin optical density (O.D.) ratios of 5 rats per group.

The density of VTA AMPARs, determined by the expression of their constitutive GluA1 subunit, showed significant differences among groups [one-way ANOVA, *F*_(2, 12)_ = 5.33, *P* = 0.02] (Figure 3A). *Post-hoc* analysis showed that the density of the GluA1 subunit decreased significantly in the sexually satiated rats. This decrease was statistically significant as compared to males ejaculating once (*P* < 0.05), and very close to significance when compared to the control group (*P* = 0.054). Interestingly, in the animals that copulated to satiety in the presence of the CB1R antagonist AM251 (Figure 3A), a significant increase in the expression of the GluA1 subunit was found as compared to the sexually satiated rats without treatment (*t*-test, *P* < 0.002), suggesting that the eCBs released during the sexual satiety development contributed to the decrease in AMPAR density.

There were changes also in GluA2 subunit expression among groups [one-way ANOVA, *F*_(2, 12)_ = 22.71, *P* < 0.001]. In this case, a significant increase in the expression of this subunit was found both in the animals ejaculating once (*P* = 0.001) and in the sexually satiated males (*P* < 0.001), when compared with control rats (Figure 3B). This increase was not modified in the males that copulated to satiety in the presence of the CB1R antagonist (Figure 3B), indicating that eCBs were not involved in this change.

### Changes in NMDARs of the VTA Induced by Copulation to Satiety and Involvement of eCBs in Their Induction

**Figure 4:**
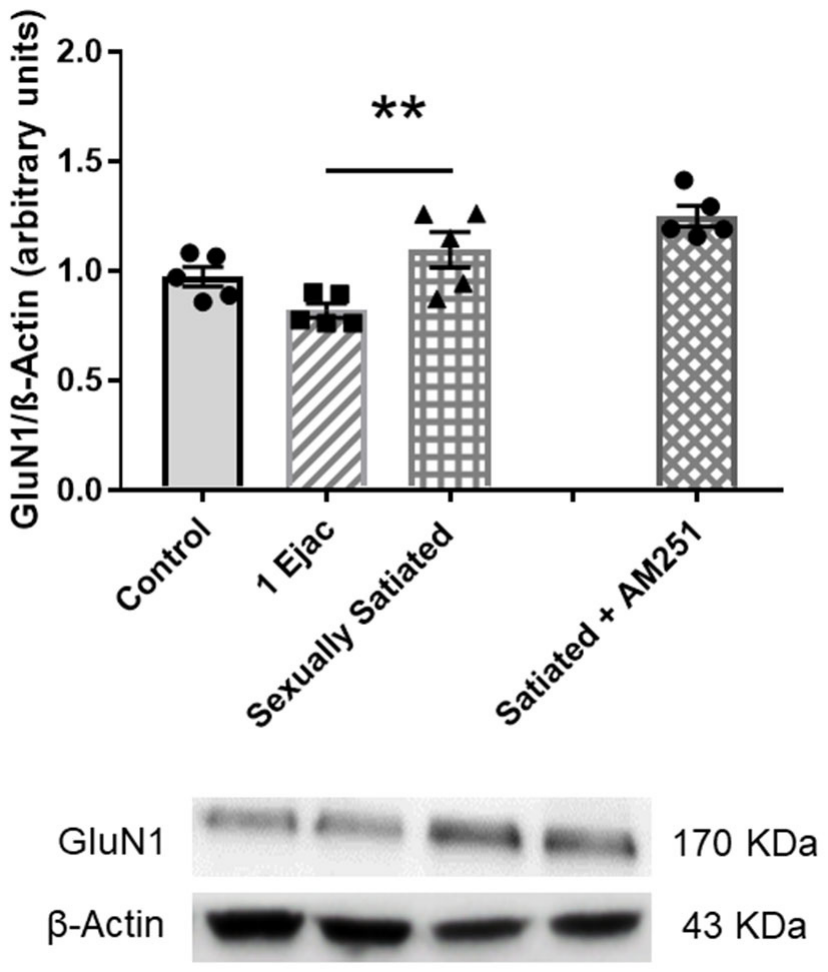
VTA NMDAR density does not change in response to sexual activity in male rats. Western blot analysis showing changes in the expression of the NMDAR constitutive GluN1 subunit in the VTA of control sexually experienced unmated rats (Control), males that ejaculated once (1 Ejac) or copulated to satiety (Sexually satiated) 24 h earlier, and of males that copulated to satiety in the presence of the CB1R antagonist AM251 (Satiated + AM251). Differences among untreated groups with different sexual conditions were determined by means of a one-way ANOVA followed by Tukey test, ^**^P < 0.01. A comparison between the rats that copulated to satiety in the absence or presence of AM251 (Satiated + AM251) was conducted with the unpaired *t*-test, n.s. Values are mean ± S.E.M of the protein/β-Actin optical density (O.D.) ratios of 5 rats per group.

Ventral tegmental area NMDAR density, determined through the measurement of its constitutive subunit GluN1, in the animals with different sexual activity showed differences among groups [one-way ANOVA, *F*_(2, 12)_ = 6.05, *P* = 0.015]. *Post-hoc* analysis indicated a statistically significant increase in GluN1 expression in the sexually satiated males only when compared to males ejaculating once (*P* = 0.012), but not in comparison with the control group (Figure 4). In the animals in which CB1Rs were blocked with AM251 during sexual satiety development, NMDAR expression was not significantly modified when compared to males copulating to satiety in the absence of treatment (Figure 4).

**Figure 5:**
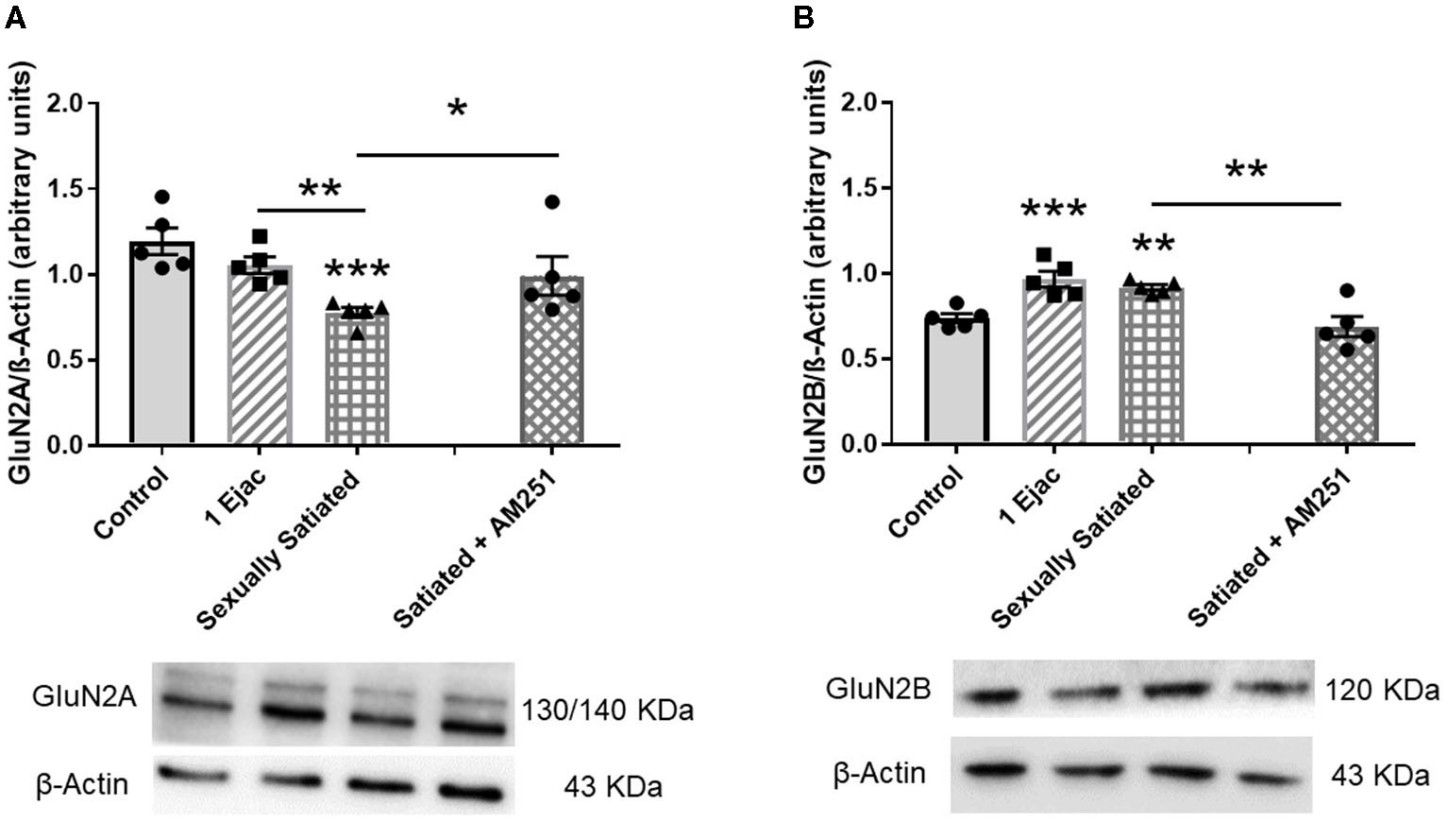
Changes in VTA NMDAR subunit composition produced by copulation to satiety in male rats and involvement of eCBs in their induction. Western blot analysis showing changes in GluN2A **(A)** and GluN2B **(B)** NMDAR subunit expression in the VTA of control sexually experienced unmated rats (Control), males that ejaculated once (1 Ejac) or copulated to satiety (Sexually satiated) 24 h earlier, and of rats that copulated to satiety in the presence of the CB1R antagonist AM251 (Satiated + AM251). Differences among untreated groups with different sexual conditions were determined by means of a one-way ANOVA followed by Tukey test, ^***^P < 0.001, ^**^P < 0.01. A comparison between the rats that copulated to satiety in the absence or presence of AM251 (Satiated + AM251) was conducted with the Mann-Whitney *U*-test, ^*^P < 0.05 or the unpaired *t*-test ^**^P < 0.01. Values are mean ± S.E.M of the protein/β-Actin optical density (O.D.) ratios of 5 rats per group.

Establishment of changes in the expression of the GluN2A and the GluN2B NMDA subunits in the distinct groups is shown in Figure 5. As it can be seen, significant changes in GluN2A subunit expression were found among groups [one-way ANOVA, *F*_(2, 12)_ = 14.3, *P* < 0.001], which was statistically significantly reduced in the sexually satiated males as compared to both control rats (*P* < 0.001) and animals ejaculating once (*P* = 0.01) (Figure 5A). Measurement of the GluN2B subunit also showed a significant effect of mating on its expression [one-way ANOVA, *F*_(2, 12)_ = 14.5, *P* < 0.001], increasing its density in both, the sexually satiated males (*P* = 0.004) and those ejaculating once (*P* < 0.001) as compared to control animals (Figure 5B). Pre-treatment with the CB1R antagonist in the animals copulating to satiety prevented the GluN2A subunit decrease (Mann–Whitney *U* test, *P* = 0.03) and the GluN2B subunit increase (*t*-test, *P* = 0.005) in the sexually satiated males (Figures 5A,B, respectively).

### Changes in pERK1/2 in the VTA Induced by Copulation to Satiety and Involvement of eCBs in Their Induction

**Figure 6:**
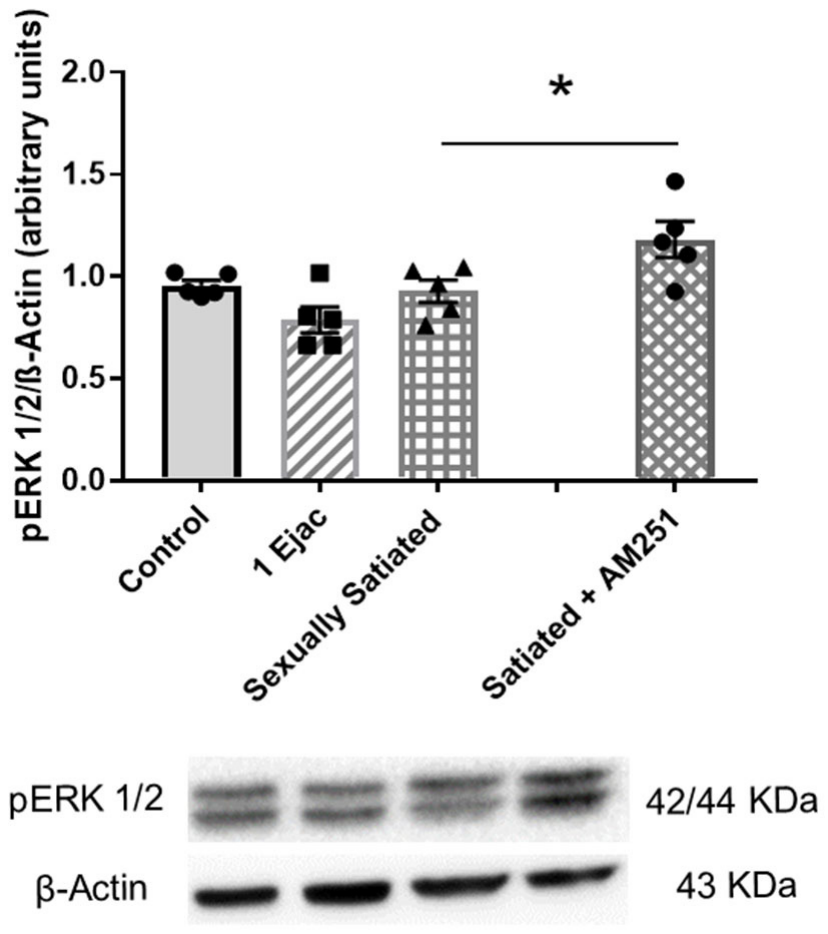
Involvement of eCBs in the changes in pERK 1/2 expression in the VTA of sexually satiated rats. Western blot analysis showing changes in pERK1/2 expression in the VTA of control sexually experienced unmated rats (Control), males that ejaculated once (1 Ejac) or copulated to satiety (Sexually satiated) 24 h earlier, and of rats that copulated to satiety in the presence of the CB1R antagonist AM251 (Satiated + AM251). Differences among untreated groups with different sexual conditions were determined by means of a one-way ANOVA, n.s. A comparison between the rats that copulated to satiety in the absence or presence of AM251 (Satiated + AM251) was conducted with the unpaired *t*-test, ^*^P < 0.05. Values are mean ± S.E.M of the protein/β-Actin optical density (O.D.) ratios of 5 rats per group.

The expression of pERK1/2 was not modified by sexual activity (Figure 6) [one-way ANOVA, *F*_(2, 12)_ = 3.12, *P* = 0.08]. However, in the males that copulated to satiety in the presence of AM251, a statistically significant increase in pERK1/2 expression was found when compared to untreated sexually satiated animals (*t*-test, *P* = 0.04), suggesting that eCBs hinder the phosphorylation of this MAPK (Figure 6).

## Discussion

Main findings of the present series of experiments are as follows: (1) copulation to satiety promotes the release of eCBs in the VTA, which activate CB1Rs and produce their internalization and downregulation; (2) 24 h after the intense sexual activity that takes place during sexual satiety development, changes in VTA glutamate AMPAR density and subunit composition and in NMDAR subunit composition are detected, coinciding with the period of sexual inhibition that characterizes sexually exhausted male rats; (3) the eCBs released during sexual activity are involved in the induction of changes in AMPAR and NMDAR, through the activation of CB1Rs, and prevent ERK1/2 phosphorylation in the sexually satiated rats.

Activation of CB1Rs at presynaptic inputs onto VTA DA neurons results from “on demand” postsynaptic eCB release during periods of increased DA neuronal activity (Lupica and Riegel, 2005). These eCBs exert control over MSL DA neuron activity by modulating GABAergic and glutamatergic inputs at the VTA (Riegel and Lupica, 2004). Sexual activity is a stimulus that enhances MSL DA neuron firing and, therefore, could promote eCB mobilization. The present study provides evidence showing that both, moderate (a single ejaculatory series) and intense copulation (until sexual satiety) activate CB1Rs at the VTA and induce their downregulation. Thus, 24 h after sexual activity, we found a significant reduction in VTA CB1R density in both copulating groups. Prolonged exposure to CB1R agonists is a condition found to produce CB1R downregulation, requiring new protein synthesis for receptor recovery (Hsieh et al., 1999). The majority of the available data on CB1R downregulation has been found following chronic tetrahydrocannabinol (THC) or synthetic cannabinoid agonist treatment (Sim-Selley, 2003), while only few studies report changes in the expression of CB1R in the brain induced by endogenous cannabinoids, and these are frequently associated with diseases involving pathological neuronal excitation. For example, a decrease in CB1R density has been documented in epileptic human hippocampal tissue (Ludányi et al., 2008), in rat hippocampus as a result of chronic stress (Hill et al., 2005; Hillard, 2014), chronic oxidative stress (Li et al., 2020), and in the ventral midbrain of patients with Parkinson's disease (Van Laere et al., 2012). The present study gives evidence for a reduction in VTA CB1R density under physiological conditions, produced by sexual activity. Little is known about the mechanisms involved in regulating the CB1R expression; again, these mechanisms have been related to disease states (Miller and Devi, 2011). Since the release of eCBs was produced by a physiological stimulus in our model, the mechanisms involved in CB1R density reduction are probably those acting in the natural receptor cycle, involving CB1R internalization and subsequent receptor degradation (Hsieh et al., 1999). The decrease in CB1R expression here reported suggests that copulation might induce a prolonged increase in eCB levels in the VTA.

No changes in the expression of phosphorylated CB1Rs (pCB1Rs) were found in any of the copulating groups of rats as compared to unmated control males. However, the lack of change in pCB1R expression, combined with the decrease in CB1R density in the VTA of these animals, implies that a larger proportion of the expressed CB1Rs is phosphorylated 24 h after sexual activity. These data strongly suggest that the proportion of pCB1Rs increases in the VTA of male rats as a result of sexual activity, thereby evidencing CB1R activation. The G protein-coupled receptor (GPCR) phosphorylation followed by binding an arrestin is a common pathway for uncoupling GPCRs, like the CB1R, from G protein-dependent signaling (Moore et al., 2007). G protein receptor kinases (GRKs) are recruited to the plasma membrane and participate in phosphorylating the agonist-occupied receptor, increasing the affinity of β-arrestins for the receptor; β-arrestin binding prevents further receptor activation. Thus, ligand-induced CB1R phosphorylation results in receptor desensitization and β-arrestin recruitment (Nogueras-Ortiz and Yudowski, 2016). β-Arrestins also work as a scaffold for the endocytic machinery that internalizes GPCRs. The recruitment of β-arrestin2 (β-A2) has been associated with the internalization process of the CB1R (Gyombolai et al., 2013). In the present study, no difference in the magnitude of VTA pCB1Rs expression was found between animals that executed mild and intense copulation the day before, though an increase in CB1R/β-A2 co-localization, interpreted as indicative of CB1R internalization, was only found in the sexually satiated rats. It has been reported that, in contrast to synthetic cannabinoid agonists, which cause rapid CB1R internalization, an analog of the endogenous cannabinoid anandamide causes internalization only at high concentrations (Hsieh et al., 1999). Thus, it could be thought that after one ejaculation, the eCBs released by DA neurons activated CB1Rs inducing their phosphorylation but did not attain the concentrations needed to induce CB1R internalization, while in the animals that copulated to satiety, DA neurons were activated repeatedly, releasing eCBs for a longer period and reaching the concentrations needed to induce CB1R internalization. Another possible interpretation for this differential result is related to the timing of CB1R internalization, since short-term agonist exposure has been reported to induce rapid CB1R internalization and recycling (Hsieh et al., 1999). This rapid process could have occurred in the animals that experienced one ejaculation and therefore, CB1R/β-A2 colocalization was no longer observed 24 h later. In contrast, the repeated activation of DA neurons during copulation to satiety could have released a larger amount of eCBs over a longer lasting period, inducing several cycles of CB1R internalization, which were still detectable 24 h later. To the best of our knowledge, this is the first time that evidence is provided for *in vivo* CB1R internalization in VTA neurons, mediated by endogenous cannabinoids that were released by a natural stimulus, under physiological conditions. In this sense, this finding is of particular significance as it provides direct information on the functioning of the endogenous cannabinoid system in the MSL circuit, specifically in the VTA, contributing to our understanding of the role of the endogenous cannabinoid system in the function of the central nervous system.

The VTA neurons that are activated by copulation in male rats, identified by the mating-induced expression of Fos protein, receive glutamatergic afferents from the medial prefrontal cortex (mPFC) (Tzschentke, 2001; Balfour et al., 2006), the pedunculopontine nucleus (PPT), and the laterodorsal tegmental (LDT) area (Clements and Grant, 1990). In addition, local glutamatergic neurons were identified in the VTA (Yamaguchi et al., 2007; Morales and Root, 2014), which form functional synapses onto VTA dopaminergic neurons (Dobi et al., 2010; Wang et al., 2015). The sex-activated VTA neurons contain NMDARs, whose constitutive GluN1 subunit is phosphorylated in response to sexual behavior and sex-associated cues (Balfour et al., 2006). Thus, glutamatergic transmission at the VTA participates in the stimulation of the MSL system that takes place during sexual activity. Besides, it has been shown that the activation of NMDARs in this brain region is necessary to enable DA neurons to switch between tonic and phasic firing patterns (Sombers et al., 2009). Both, sexual cues and mating itself, trigger mesolimbic DA neuron phasic firing, resulting in an increase in DA release at the NAcc (Pfaus et al., 1990; Damsma et al., 1992; Wenkstern et al., 1993; Robinson et al., 2001). Together, these data show that glutamatergic synapses onto DA cell bodies in the VTA play an important role in the response of MSL DA neurons to sexual activity, a natural rewarding stimulus (Floresco et al., 2003).

In the present work, we provide evidence for changes in VTA glutamate receptor density and subunit composition that were detected in the sexually satiated rats 24 h after intense sexual activity. Thus, a decrease in the density of VTA AMPARs, concomitant to a significant rise in the expression of AMPARs containing the GluA2 subunit were found in the sexually satiated rats. Repeated activation of Ca^2+^- permeable AMPARs has been reported to produce a long-lasting switch in their subunit composition, from GluA2-lacking to GluA2-containing receptors (Liu and Cull-Candy, 2000). Thus, the increased expression of the GluA2 subunit found in the sexually satiated males, suggests that the proportion of GluA2-containing AMPARs was increased as a result of the repeated activation of VTA neurons by glutamate during copulation to satiety. It is well-established that the presence of the GluA2 subunit in AMPARs renders these receptors impermeable to Ca^2+^ and Zn^2+^ and slows the channel kinetics (Liu and Zukin, 2007). Therefore, the predominance of GluA2-containing AMPARs results in a reduction of synaptic activity. On the other side, the decrease in the number of AMPAR may result from their removal from the cell surface by internalization and their subsequent lysosomal degradation. This phenomenon is associated with a specific form of long-term plasticity: long-term depression (LTD) (Chater and Goda, 2014). LTD of excitatory synapses can be produced by a switch in the composition of AMPARs from GluA2-lacking to GluA2-containing receptors, which show a lower single channel conductance (Mameli et al., 2007), and requires a loss of AMPARs from the synapse (Chater and Goda, 2014). The decrease in AMPAR expression and the increase in GluA2-containing receptor expression observed in this work in the sexually satiated rats are in line with those producing LTD.

As to the NMDAR, its density in the VTA was increased in the sexually satiated animals as compared to males that ejaculated only once but was not importantly modified with respect to control animals, in contrast to the evident changes found in the expression of NMDAR subunits. In this last case, a significant decrease in the expression of the GluN2A subunit, concomitant to a significant increase in that of the GluN2B subunit were found in the VTA of the sexually exhausted males, suggesting that the subunit composition of NMDARs is changed. Such changes in NMDAR composition would be relevant, as the GluN2A subunit confers these receptors a lower affinity for glutamate, faster kinetics, and greater channel open probability (Lau and Zukin, 2007). Synaptic activity regulates the molecular composition of NMDARs in such a manner that there is a relative abundance of GluN2A containing receptors in active synapses, which shifts to a reduction relative to the abundance of GluN2B containing NMDARs in inactive synapses (Ehlers, 2003). Therefore, a presumed increase in the proportion of GluN2B-NMDARs, with slower kinetics and reduced channel open probability, with regard to GluN2A-NMDARs, characterized by a high channel open probability and rapid deactivation (Paoletti et al., 2013), would result in a reduced activity of the neurons in which these changes occur. It could be speculated that the changes in expression of NMDAR subunits found in the VTA of the sexually satiated rats, might indicate a reduced activity of VTA glutamatergic synapses during the sexual inhibitory state of these animals. An increase in GluN2B subunit expression was also found in the males that ejaculated once, suggesting that glutamatergic stimulation provided by mild sexual activity is sufficient to initiate changes in NMDAR subunit composition.

We found only one study investigating changes in the expression of glutamate ionotropic receptors associated with male sexual activity in the MSL system. In that work, an increase in NMDAR density without changes in the expression of GluA1 and GluA2 AMPAR subunits was found in the NAcc of male rats, 24 h after the last of five consecutive daily mating sessions involving one ejaculation per day (Pitchers et al., 2012). Together with present findings, these data show that a natural rewarding behavior like sexual activity can induce changes in glutamate receptor expression in the MSL system.

Sexually satiated male rats do not respond to the presence of a sexually receptive female 24 h after copulation to satiety. This is an unexpected behavior since, on the one side, these animals are proofed sexually competent rats that rested around 20 h after intense sexual activity when presented to the new sexual partner and, on the other, copulation is an instinctive behavior that should be triggered by an adequate sign stimulus: in this case, the sexually receptive female. Considering that glutamatergic stimulation at the VTA participates in changing the activity pattern of DA neurons toward phasic firing, a phenomenon that occurs in response to rewarding stimuli, changes in the subunit composition of NMDARs, might reduce or cancel the ability of DA neurons to produce this phasic firing, thereby contributing to the lack of response of the sexually satiated rats to the presence of a sexually receptive female. Though the neuronal composition of the VTA is heterogeneous and, in addition to DA neurons (65%), there is an important population of GABA neurons (around 30%) and a small proportion of glutamate neurons (around 5%) (Dobi et al., 2010; Morales and Margolis, 2017). These GABA neurons exhibit NMDAR-mediated plasticity (Nelson et al., 2018) and can therefore be both, targets of glutamate and contribute indirectly to the induction of changes in DA neuron firing activity (Bouarab et al., 2019).

One of the goals of the present study was to determine the possible participation of the eCBs released during copulation to satiety in the changes in glutamatergic transmission resulting from intense sexual activity. Our results show that the blockade of the actions of eCBs at CB1R during copulation to satiety, by the pretreatment with the CB1R antagonist AM251, interfered with the decrease in AMPAR density and blocked the changes in the expression of NMDAR subunits, but did not modify the changes in the subunit composition of AMPARs. The question emerges as to the mechanisms involved in these eCB-induced changes in glutamatergic receptors.

It has been shown that prolonged DA neuron depolarization within the VTA induces the release of eCBs, which activate CB1Rs at glutamatergic presynaptic terminals suppressing glutamatergic transmission onto DA neurons (Melis, 2004), through the short-term plasticity phenomenon called DSE (Kreitzer and Regehr, 2001). It has been considered that DSE plays a neuroprotective role against glutamate-induced excitotoxicity (Diana and Marty, 2004). The changes in VTA glutamatergic receptors reported here were detected particularly in the sexually satiated rats and are therefore consistent with a longer or repeated glutamatergic receptor stimulation. The eCBs have been found to produce also long-term plasticity phenomena, like long-term depression (eCB-LTD) of glutamatergic transmission (Robbe et al., 2002), requiring either long or repeated CB1R activation to be induced (Diana and Marty, 2004). This eCB-LTD has been observed in VTA DA neurons and is produced by a decrease in glutamate release, presumably exerted by 2-AG through the activation of CB1Rs (Haj-Dahmane and Shen, 2010). This form of plasticity is mechanistically different from the eCB-mediated mGluR-LTD, also described in the VTA (Bellone and Lüscher, 2005), in which the postsynaptic group I mGluR activation, triggers the synthesis and release of 2-AG, presumed to mediate a switch in AMPAR subunit composition to induce LTD. The excitatory synapses onto DA neurons of the VTA contain GluA2-lacking AMPARs and mGluR-LTD induces an increase in the intracellular calcium of DA neurons (Morikawa et al., 2003) leading to eCB release (Wilson and Nicoll, 2002) that depend on the switch from GluA2-lacking to GluA2-containing AMPARs. These two forms of LTD of glutamatergic synapses on DA neurons of the VTA coexist and might be part of the mechanisms contributing to the induction of the long-lasting physiological changes observed in sexually satiated rats. We have demonstrated that interfering with CB1R activation during sexual satiety development hinders the appearance of the long-lasting sexual behavior inhibition and drug hypersensitivity in rats that copulate to satiety (González-Morales and Rodríguez-Manzo, 2020). Blockade of CB1Rs during intense copulation could have interfered with eCB-LTD but not with mGluR-LTD, since the released glutamate would have activated mGluRs. Present data showing that CB1R blockade during copulation to satiety did not cancel the increase in GluA2-containing AMPARs support this notion.

Although eCB-mediated LTD plasticity phenomena may play a role in the induction of the long-lasting plastic changes that characterize sexually exhausted rats, their dependence on CB1R activation makes eCB-LTD the most likely candidate. Electrophysiological studies should be conducted to evaluate this possibility.

Extracellular signal-regulated receptor kinase (ERK) participates in the induction of some forms of LTD (Gallagher et al., 2004; Grueter et al., 2006; Kellogg et al., 2009) and cannabinoid receptors are known to activate ERK (Derkinderen et al., 2003); therefore, we decided to look for changes in ERK phosphorylation in the VTA of sexually satiated rats that might be related to CB1R activation. Our data failed to detect changes in pERK1/2 expression in the VTA of the copulating males; however, blockade of eCB actions during intense copulation resulted in an increase in VTA pERK1/2 expression in the sexually satiated males, suggesting that the released eCBs hinder the increase in the phosphorylation of this MAP kinase. Contrary to present results, CB1R-mediated ERK1/2 phosphorylation has been shown to occur and to participate in eCB-dependent LTD at inhibitory synapses (I-LTD) onto VTA DA neurons, as a result of repeated cocaine exposure (Pan et al., 2011). We could not find any data on CB1R-mediated ERK1/2 phosphorylation at excitatory synapses; however, it has been reported that sustained CB1R stimulation activates ERK1/2 only transiently and that the duration of ERK1/2 activation is regulated by CB1R desensitization (Daigle et al., 2008). However, in our study the observed increase in pERK1/2 expression in the AM251 pre-treated males did not involve CB1R activation but must have been the result of stimulating other type of receptors, for instance, NMDA glutamate receptors (Krapivinsky et al., 2003).

In sum, the results of the present work show that eCBs are released in the VTA in response to both mild and intense sexual activity; however, only in the rats that copulated to satiety did eCBs induce excitatory synaptic plasticity. These data suggest that the magnitude of eCB release was different in these two groups. Copulation to satiety appears to have released a larger amount of eCBs in the VTA, which impacted glutamatergic transmission presumably onto DA neurons, although VTA GABA neurons expressing glutamate receptors could also be a target. The nature of the glutamate receptor changes promoted by eCBs is consistent with a reduction in the excitatory effects of glutamatergic input, which might result in a reduced VTA DA and maybe also GABA neuron activity. The present work provides evidence for a physiological role of eCB release from VTA DA neurons *in vivo*, that might represent a protective mechanism against excessive activation produced by heightened natural rewarding stimulation.

## Conclusion

Evidence implicates eCBs in the induction of the long-lasting physiological changes observed in sexually satiated rats: the sexual inhibition and generalized sensitization to drug actions. The MSL system, specifically the VTA, has been found to play a significant role in the control of these two phenomena (Canseco-Alba and Rodríguez-Manzo, 2014; González-Morales and Rodríguez-Manzo, 2020). CB1Rs on axon terminals synapsing onto DA neurons in this brain region are the target of these eCB-mediated actions and glutamatergic transmission appears to be a principal player. The results of the present series of experiments demonstrate that eCBs are released in the VTA during copulation to satiety and activate CB1Rs producing changes in glutamate receptors that are consistent with a reduction of glutamate excitatory effects onto DA and perhaps GABA neurons.

## Data Availability Statement

The raw data supporting the conclusions of this article will be made available by the authors, without undue reservation.

## Ethics Statement

The animal study was reviewed and approved by Comité Institucional para el Cuidado y Uso de Animales de Laboratorio (CICUAL) at CINVESTAV.

## Author Contributions

GR-M conceived and designed the study and wrote the manuscript. EG-M and RG-G performed the experiments and participated in the drafting of the manuscript. EG-M prepared the figures. All authors analyzed the data, discussed the results, and commented on the manuscript.

## Conflict of Interest

The authors declare that the research was conducted in the absence of any commercial or financial relationships that could be construed as a potential conflict of interest.

## Publisher's Note

All claims expressed in this article are solely those of the authors and do not necessarily represent those of their affiliated organizations, or those of the publisher, the editors and the reviewers. Any product that may be evaluated in this article, or claim that may be made by its manufacturer, is not guaranteed or endorsed by the publisher.
